# Age at job initiation and risk of coronary heart disease: findings from the UK biobank cohort study

**DOI:** 10.1186/s12889-023-17034-3

**Published:** 2023-10-30

**Authors:** Zenghui Zhang, Chuanrui Zeng, Zhiteng Chen, Pinming Liu, Jingwei Gao, Qi Guo, Maoxiong Wu, Wanbing He, Qingyuan Gao, Dachuan Guo, Xiaotian Liang, Zegui Huang, Jingfeng Wang, Haifeng Zhang, Yangxin Chen

**Affiliations:** 1 Department of Cardiology, Sun Yat-sen Memorial Hospital, Sun Yat-sen University, Guangzhou, 510120 China; 2 Guangdong Province Key Laboratory of Arrhythmia and Electrophysiology, Sun Yat-sen Memorial Hospital, Sun Yat-sen University, Guangzhou, China; 3 Guangzhou Key Laboratory of Molecular Mechanism and Translation in Major Cardiovascular Disease, Sun Yat- sen Memorial Hospital, Sun Yat-sen University, Guangzhou, China

**Keywords:** Young worker, Risk factor, Coronary heart disease, Early work exposure

## Abstract

**Background:**

Commencing work at an early age has been linked to various risk factors for coronary heart disease (CHD), such as shift work and intensive job strain. However, the relationship between starting work too early and CHD risk remains largely unclear. We examined the association between age at job initiation and the risk of CHD.

**Methods:**

UK Biobank participants aged 38 to 70 years without cardiovascular disease who provided data on their age at job initiation were included. The primary outcome was CHD, which was ascertained using hospital and death records. The hazard ratios (HRs) and 95% confidence interval (CIs) for the association between age at job initiation and CHD were calculated using multivariable Cox regression.

**Results:**

Of the 501,971 participants, 114,418 eligible participants were included in the final analysis. The median age at job initiation was 19.0 years. During the mean follow-up of 12.6 years, 6,130 (5.4%) first CHD events occurred. We observed that age at job initiation was inversely associated with CHD (HR 0.98, 95% CI 0.97–0.99), and the association was potentially J-shaped. The HRs for the < 17-year, 17–18-year, and 19–21-year age groups were 1.29 (95%CI 1.18–1.41), 1.12 (95% CI 1.03–1.22) and 1.05 (95% CI 0.97–1.14), respectively, compared with those of the ≥ 22-year group.

**Conclusions:**

Age at job initiation was associated with incident CHD, which was independent of socioeconomic status. Participants who commenced employment before the age of 19 years exhibited a higher risk of developing CHD later in adulthood.

**Supplementary Information:**

The online version contains supplementary material available at 10.1186/s12889-023-17034-3.

## Background

Coronary heart disease (CHD) is a highly prevalent atherosclerotic cardiovascular disease (CVD) that exerts a substantial and enduring global burden on public health [1, 2]. Despite advancements in the control of modifiable risk factors, the morbidity and mortality of CHD remain high. Notably, Chinese epidemiological data revealed approximately 8.9 million deaths and 164.0 million disability-adjusted life years were attributed to CHD in 2015 [3]. This epidemiological picture underscores the critical need to uncover novel risk factors to bolster CHD prevention efforts.

In addition to traditional risk factors such as smoking, hyperlipidemia, and obesity, several occupational risk factors—job strain and shift work being notable examples— have emerged as contributors to the elevated risk for CHD [4, 5]. Importantly, however, the risk of CHD exists even among workers who are not subjected to these known hazards, suggesting potential unrecognized job-related risk factors that may play a role in mediating the effects of work on the risk of CHD. Of particular interest are young workers, a significant segment of the labor force that shoulders the burgeoning demands of contemporary social production [6, 7]. Recent data from the United Kingdom’s Office for National Statistics revealed that young workers aged 16–17 years made up a substantial (35.3%) proportion of the employment rate in 2022 [7]. Moreover, a report on occupational injuries among young workers in the United States unveiled this group’s heightened susceptibility to job-related injuries and illnesses compared with their adult counterparts [6]. This heightened vulnerability can be attributed to factors including the incorrect use of tools and equipment, emotional immaturity, and inappropriate judgment in emergencies [8–11].

Importantly, a significant number (approximately 3.2 million) of nonfatal, job-related injuries occurred among worker aged 15–24 years in the United States [6]. Within this age group, workers aged 18–19 years experienced the highest injury rates and necessitated treatment in hospital emergency departments. Adding to these concerns, young workers under the age of 19 years often work long shifts, including intensive night shifts that extend past 9 p.m., especially during school years [8, 12]. This situation leads to increased emotional stress and the adoption of unhealthy behaviors such as substance abuse, smoking, and alcohol consumption [12]. Moreover, the initiation of work at an early age diverts the focus of young workers from educational pursuits [13, 14].

A significant proportion of young workers face elevated levels of work-related stress alongside notable risks of both fatal and non-fatal occupational hazards [6, 15–17]. These challenges, coupled with the potential for emotional distress and the adoption of unhealthy lifestyles [12], underscore the potential risks of commencing work at an early age. Multiple studies have established associations between work-related stress [18], emotional distress [19], unhealthy lifestyles [20], and a heightened risk of CHD. However, the association between early work exposure and CHD risk remains unknown. Hence, the primary objective of this study is to investigate the relationship between the age at which individuals initiate their employment and the risk of developing CHD.

## Method

### Study design and participants

We obtained data from the UK Biobank, a large prospective cohort study consisting of more than 500,000 participants recruited from twenty-two assessment centers across the United Kingdom between 2006 and 2010 [21]. The detailed information on questionnaires, ethical approvals, and data access policies for UK Biobank are available online. UK Biobank received ethical approval from the National Information Governance Board for Health and Social Care and the National Health Service North West Multi-Center Research Ethics Committee. All written informed consent were provided from participants before enrollment in the study. A flowchart of detailed inclusion and exclusion of participants was available in supplementary material online.

### Assessment of age at job initiation

Participants were invited to complete the questionnaires on their employment history using a web-based recall questionnaire. After specifying their setup information, participants were presented with a table which allowed them to enter job and gap periods to build up their lifetime history. The information on jobs/gaps were in date order. The exact age at job initiation were obtained by the year of birth minus the year of job started.

### Ascertainment of coronary heart disease

Due to the unavailability of the 11th International Classification of Diseases (ICD-11) within UK Biobank dataset, incident cases of CHD were defined based on the criteria outlined in the International Classification of Diseases, 10th Revision (ICD-10), specifically encompassing codes I20 to I25 (Additional file 1: Table S1). Detailed information regarding the diagnosis of CHD and baseline CVD can be found in the Table S1. Within the UK Biobank cohort, participants were diligently followed up until the occurrence of their first CHD event, mortality, or the conclusion of the follow-up period.

### Assessment of covariates

Covariates in this study encompassed various demographic, socioeconomic, lifestyle, health-related factors. Demographic variables included age, sex, and ethnicity (white or other). The Townsend Deprivation Index (TDI) was used to assess the level of socioeconomic disadvantage in specific geographic areas, considering factors such as unemployment, housing conditions, and ownership, with higher TDI scores indicating greater deprivation. Socioeconomic factors incorporated household income, categorized as low (<£18,000), middle (£18,000-£100,000), or high (>£100,000), and educational attainment (college or university degree). Lifestyle variables consisted of obesity (body mass index ≥ 30 kg/m²), drinker (current or previous drinking) and smoker (current or previous smoking), and physical activity levels (≥ 150 min/week of moderate intensity, ≥ 75 min/week of vigorous intensity, or an equivalent combination). Dietary patterns were classified as unhealthy if the intake of fruits and vegetables was below the median and the intake of red meat, processed food, and added salt was above the median; healthy if the reverse was true; and intermediate for the remaining individuals [22]. Employment-related variables included shift work, work category (managerial, professional, technical, skilled trades, or other occupations), and work hours per week (15 to less-than-20 h, 20 to less-than-30 h, 30 to 40 h, over 40 h).

Mental health factors in the study encompassed various aspects, including whether individuals had sought psychiatric help for conditions related to nerves, anxiety, tension, or depression. Additionally, participants were assessed for feelings of worry, anxiety, sensitivity, hurt, and experiences of loneliness or isolation. These mental health variables were gathered through self-reported questionnaires, with participants responding to questions such as “Have you ever seen a psychiatrist for nerves, anxiety, tension, or depression?“, “Are you a worrier?”, “Are your feelings easily hurt?”, and “Do you often feel lonely?“. If participants responded affirmatively with a “Yes,“ they were categorized as having a positive response in relation to these mental health factors.

Health-related variables covered ideal systolic blood pressure (< 120 mmHg), ideal diastolic blood pressure (< 80 mmHg), hyperlipidemia, and diabetes mellitus. Covariates used in sensitivity analysis included family history of CVD (presence of stroke or heart disease in parent or siblings), chronic stress as reflected by the Patient Health Questionnaire-4 (PHQ4) questionnaire score, and chronic kidney disease. The PHQ-4 score, ranging from 0 to 12, summarizes participants’ responses to four items assessing the frequency of depressed mood, unenthusiasm/disinterest, tenseness/restlessness, and tiredness/lethargy [23]. Detailed information about missing value can be found in the supplementary materials online (Additional file 1: Table S2). To address these missing values, we performed multiple imputations using chained equations. This approach involved creating 5 datasets using the MICE R package, a widely used method that considers the uncertainty associated with missing data [24].

### Statistical analysis

All analysis were performed by R version 4.1.3. a *P* < 0.05 was considered statistically significant. We described participants’ baseline characteristics with mean (standard deviation) and number (percentage) for continuous and categorical variables respectively. Participants’ baseline characteristics were compared by analysis of variance for continuous and χ2 test for categorical variables.

The multivariate Cox proportional hazards regression was established to calculate the hazard ratios (HRs) and 95% confidence intervals (CIs) for the association between the age at job initiation with incidence of CHD. Two models were employed for adjustment: Model1: Adjusted for age and sex; Model2: Adjusted for age, sex, ethnicity; obesity; TDI; household income; college or university degree; smoking status; drinking status; physical activity; diet quality; work category; presence of shift work; work hours per week; history of seeing a psychiatrist for nerves, anxiety, tension or depression; feelings of worry/anxiety, sensitivity/hurt, or loneliness isolation; ideal systolic and diastolic blood pressure; hyperlipemia; and diabetes mellitus. In order to model potential nonlinear relationships, nonparametrically restricted cubic spline regression was performed with knots at the 25th, 50th, and 75th percentiles between the age at job initiation and incidence of CHD. To investigate this association, we categorized the age at job initiation, according to quantile, into the following four groups: < 17 years, 17–18 years, 19–21 years and ≥ 22 years respectively. Among these group, ≥ 22 years group was used as reference group. Kaplan-Meier analysis was used to evaluate the cumulative risk of CHD in each group above. Subgroup analysis was performed according to stratification by covariates and we calculated the *p*-values for interaction based on a log likelihood ratio test comparing models with and without cross-product interaction terms.

### Sensitivity analysis

Several sensitivity analyses were performed to assess the robustness of our findings: (1) Exclusion of Recent CHD Cases: Participants who had experienced CHD within 2 years after follow-up were excluded to account for potential reverse causality. (2) Outlier Exclusion: Participants with age at job initiation classified as outliers (< 9.5 years old or > 29.5 years old based on the interquartile range) were excluded to mitigate the influence of extreme values. (3) Competing Risk Analysis: Death was considered a competing risk event rather than a censoring event, and a competing risk model was employed to assess the association between age at job initiation and endpoints. (4) Inclusion of Additional Risk Factors: Additional risk factors associated with coronary heart disease, such as family history of CVD, chronic stress as indicated byPHQ-4 score, and chronic kidney disease, were included in the analysis to account for their potential impact.

## Results

### Baseline characteristics of participants

**Fig. 1:**
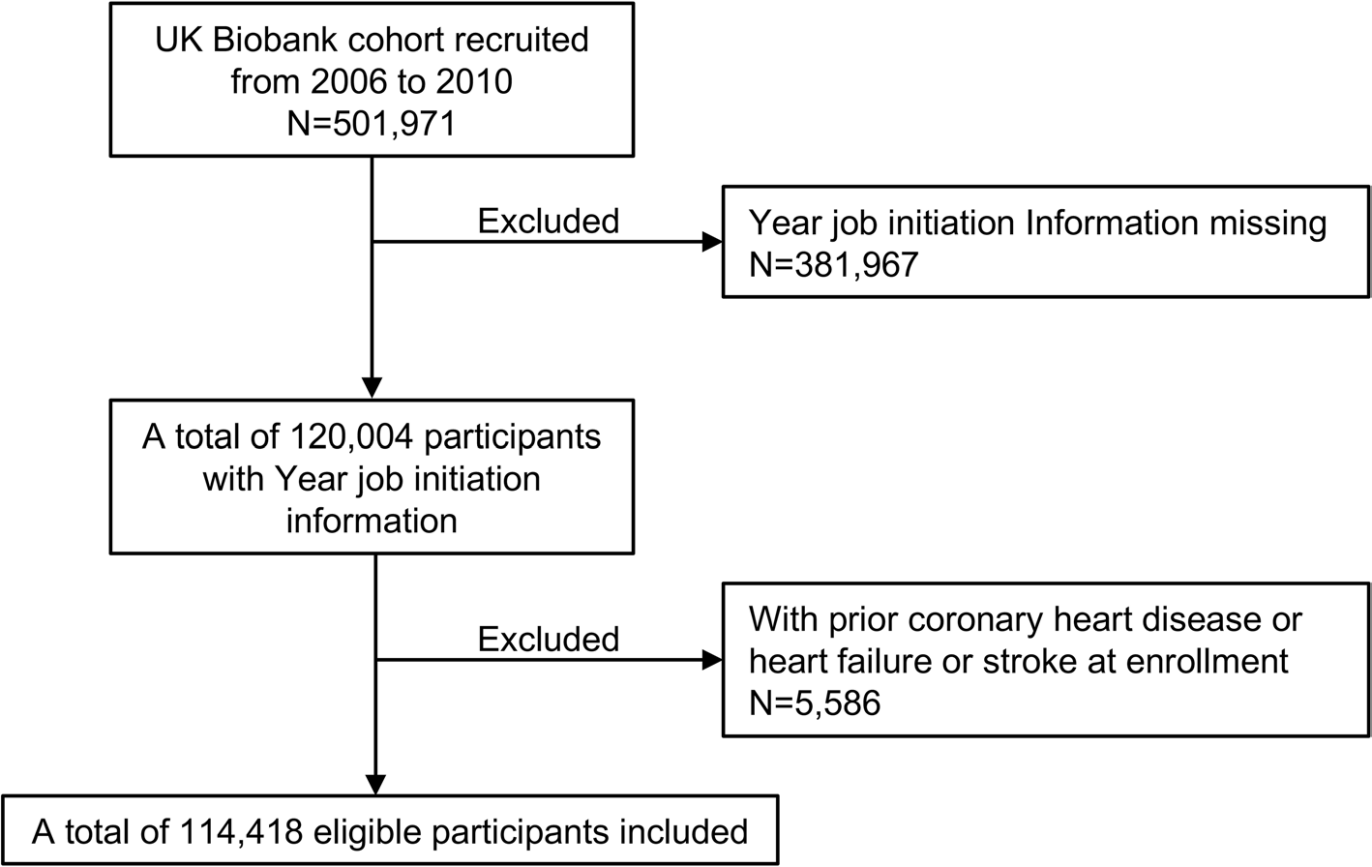
Study flowchart

After excluding participants who did not provide information on the year in which they started working (n = 381,967) and those with CVD at enrollment (n = 5,586), we included 114,418 people in the final analysis (Fig. 1). The participants had a mean age at baseline of 55.8 years (standard deviation: 7.7 years), and 49,436 (43.2%) participants were male and 64,982 (56.8%) were female. The median age at job initiation was 19.0 years (IQR 17.0–22.0); Additional file 1: Figure S1). During the mean follow-up of 12.6 years (standard deviation: 1.8 years), 6,130 CHD events (5.4%) occurred in the eligible participants. Those with CHD had a significantly younger age at job initiation compared with those without (mean 18.9 vs. 19.6 years; Additional file 1: Table S3).

### Association between age at job initiation and CHD outcomes

**Fig. 2:**
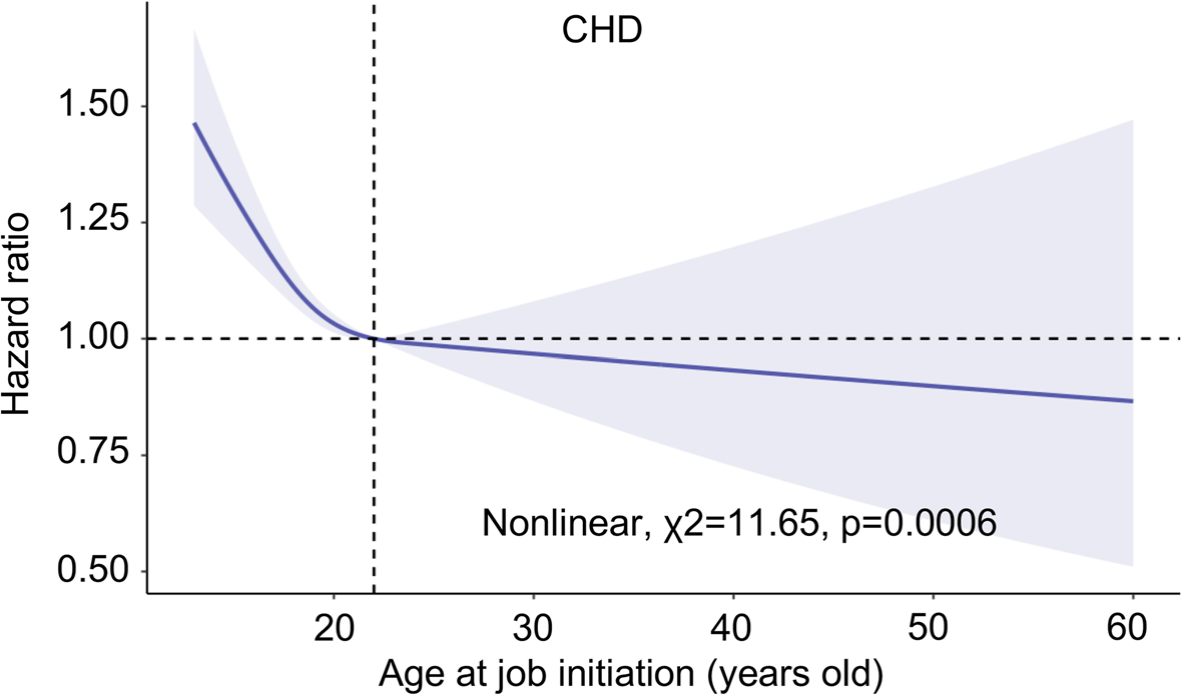
Smoothing spline plots of age at job initiation and coronary heart disease.  Note: All analysis were fully adjusted for covariates presented in Table 1. The solid blue line represents the best-fit line, and the darked area are 95% confidence intervals; CHD = coronary heart disease

In a Cox regression model that was adjusted for age; sex; ethnicity; obesity; TDI; household income; college or university degree; smoking status; drinking status; physical activity; diet quality; work category; presence of shift work; work hours per week; history of seeing a psychiatrist for nerves, anxiety, tension or depression; feelings of worry/anxiety, sensitivity/hurt, or loneliness or isolation; ideal systolic and diastolic blood pressure; hyperlipemia; and diabetes mellitus, an inverse association was observed between age at job initiation and CHD risk (HR 0.98, 95% CI 0.97–0.99; Additional file 1: Table S4). A potential non-linear relationship was noted between age at job initiation and CHD risk (*p* < 0.001; Fig. 2).

**Table 1:** Baseline participant characteristics by quantile of age at job initiation

	Age at job initiation (years old)				
	Q1 (≥ 22)	Q2 (19–21)	Q3 (17–18)	Q4 (< 17)	*P* value
n (%)	36,607 (32.0)	23,259 (20.3)	28,007(24.5)	26,545(23.2)	
Age (years)	54.9 (7.73)	55.1 (7.58)	55.4 (7.74)	58.2 (7.03)	< 0.001
Male gender, n (%)	17,074 (46.6%)	8,774 (37.7%)	11,066 (39.5%)	12,522 (47.2%)	< 0.001
White, n (%)	35,089 (95.9%)	22,595 (97.1%)	27,406 (97.9%)	26,250 (98.9%)	0.398
TDI	-1.68 (2.84)	-1.96 (2.69)	-1.96 (2.67)	-1.76 (2.73)	0.019
Household income, n (%)					< 0.001
Low	2,226 (6.08%)	1,988 (8.55%)	3,594 (12.8%)	5,769 (21.7%)	
Middle	29,106 (79.5%)	19,219 (82.6%)	23,159 (82.7%)	20,264 (76.3%)	
High	5,275 (14.4%)	2,052 (8.82%)	1,254 (4.48%)	512 (1.93%)	
Obesity	5,198 (14.2%)	4,098 (17.6%)	6,010 (21.5%)	6,494 (24.5%)	< 0.001
College or university degree, n (%)	32,721 (89.4%)	12,557 (54.0%)	6,232 (22.3%)	3,443 (13.0%)	< 0.001
Drinker, n (%)	35,673 (97.4%)	22,638 (97.3%)	27,249 (97.3%)	25,795 (97.2%)	0.207
Smoker, n (%)	12,810 (35.0%)	8,610 (37.0%)	12,087 (43.2%)	13,126 (49.4%)	< 0.001
Physical activity at goal, n (%)	30,077 (82.2%)	18,973 (81.6%)	22,687 (81.0%)	21,846 (82.3%)	< 0.001
Diet quality, n (%)					< 0.001
Healthy diet	6,699 (18.3%)	4,232 (18.2%)	4,757 (17.0%)	4,228 (15.9%)	
Intermediate diet	21,349 (58.3%)	13,294 (57.2%)	15,870 (56.7%)	15,183 (57.2%)	
Unhealthy diet	8,559 (23.4%)	5,733 (24.6%)	7,380 (26.4%)	7,134 (26.9%)	
Job involved shift work, n (%)	4,405 (12.0%)	3,584 (15.4%)	47,90 (17.1%)	3,620 (13.6%)	< 0.001
Work category, n (%)					< 0.001
Manager or administrative occupations	5,803 (15.9%)	6,404 (27.5%)	11,801 (42.1%)	8,969 (33.8%)	
Professional, technical or skilled trades occupations	28,301 (77.3%)	13,938 (59.9%)	10,854 (38.8%)	9,756 (36.8%)	
Other occupations	2,503 (6.84%)	2,917 (12.5%)	5,352 (19.1%)	7,820 (29.5%)	
Work hours per week, n (%)					< 0.001
15 to less-than-20 h	755 (2.06%)	341 (1.47%)	294 (1.05%)	217 (0.82%)	
20 to less-than-30 h	1,781 (4.87%)	988 (4.25%)	992 (3.54%)	735 (2.77%)	
30 to 40 h	19,521 (53.3%)	14,909 (64.1%)	20,073 (71.7%)	17,501 (65.9%)	
Over 40 h	14,550 (39.7%)	7,021 (30.2%)	6,648 (23.7%)	8,092 (30.5%)	
Seen a psychiatrist for nerves, anxiety, tension or depression, n (%)	3,603 (9.84%)	2,241 (9.63%)	2,589 (9.24%)	2,409 (9.08%)	0.004
Worrier / anxious feelings, n (%)	19,551 (53.4%)	12,567 (54.0%)	15,006 (53.6%)	13,950 (52.6%)	0.008
Sensitivity / hurt feelings, n (%)	17,974 (49.1%)	12,082 (51.9%)	15,137 (54.0%)	14,575 (54.9%)	< 0.001
Loneliness, isolation, n (%)	4,805 (13.1%)	3,385 (14.6%)	4,435 (15.8%)	4,177 (15.7%)	< 0.001
SBP < 120 mmHg, n (%)	7,152 (19.5%)	4,202 (18.1%)	4,512 (16.1%)	3,109 (11.7%)	< 0.001
DBP < 80 mmHg, n (%)	15,974 (43.6%)	9,798 (42.1%)	11,082 (39.6%)	9,812 (37.0%)	< 0.001
Hyperlipemia, n (%)	3,974 (10.9%)	2,511 (10.8%)	3,505 (12.5%)	4,484 (16.9%)	< 0.001
Diabetes mellitus, n (%)	869 (2.37%)	707 (3.04%)	875 (3.12%)	1,074 (4.05%)	< 0.001

Data are presented by mean (standard deviation) or n (%) for continuous and categorical variables, as appropriate. *TDI *Townsend deprivation index, *SBP *Systolic blood pressure, *DBP *Diastolic blood pressure

To further investigate the role of age at job initiation, we categorized participants into four groups by age quantile (≥ 22 years, 19–21 years, 17–18 years and < 17 years). Compared with the ≥ 22-year group, participants who started their jobs at age 17–18 years and < 17 years were more likely to be older, male gender, obese, and smokers at baseline. These participants also had a low household income, low educational attainment, unhealthy diets, jobs involved shift work, and experienced sensitivity/ hurt feelings, loneliness/social isolation, and unhealthy health status (e.g., abnormal blood pressure, hyperlipemia, and diabetes mellitus; Table 1).

A higher incidence of CHD was observed in participants who began jobs started in their adolescence (5.14% and 7.75% in those aged 17–18 years and < 17 years, respectively), compared with the adult reference (4.38% and 4.42% in those aged ≥ 22 years and 19–21 years, respectively). The cumulative risk of CHD significantly decreased from the < 17-year group to the ≥ 22-year group (Additional file 1: Figure S2). Compared with the ≥ 22-year group, age and sex-adjusted HRs for CHD risk were 1.10 (95% CI 1.02–1.19), 1.22 (95% CI 1.14–1.31) and 1.49 (95% CI 1.40–1.59) for 19–21-year, 17–18-year, and < 17-year groups respectively. In the fully adjusted Cox regression mode, which accounted for all of the covariates as presented in Table 1, the association between age at job initiation and CHD remained highly statistically significant for participants who started working before 19 years of age, with HRs of 1.12 (95% CI 1.03–1.22) for those aged 17–18 years and 1.29 (95% CI 1.18–1.41) for those aged < 17 years (Table 2).

**Table 2:** The association between age at job initiation and coronary heart disease

	Age at job initiation (years old)				
Endpoints	Q1 (≥ 22)	Q2 (19–21)	Q3 (17–18)	Q4 (< 17)	*P* for trend
CHD					
Cases n (%)	1,604 (4.38%)	1,028 (4.42%)	1,440 (5.14%)	2,058 (7.75%)	
Model1	1.00 (reference)	1.10 (1.02–1.19)	1.22 (1.14–1.31)	1.49 (1.40–1.59)	< 0.001
Model2	1.00 (reference)	1.05 (0.97–1.14)	1.12 (1.03–1.22)	1.29 (1.18–1.41)	< 0.001

model1 adjusted for age and sex; model2 adjusted for covariates on model1 plus ethnicity, obesity, Townsend deprivation index, household income, college or university degree, smoking status, drinking status, physical activity, diet quality, work category, shift work, work hours per week, seen a psychiatrist for nerves, anxiety, tension or depression, worrier/anxious feeling, sensitivity/hurt feeling, loneliness isolation, systolic blood pressure<120 mmHg, diastolic blood pressure<80 mmHg, hyperlipemia, diabetes mellitus; *CHD *Coronary heart disease

### Interaction between covariates and subgroup analysis

**Fig. 3:**
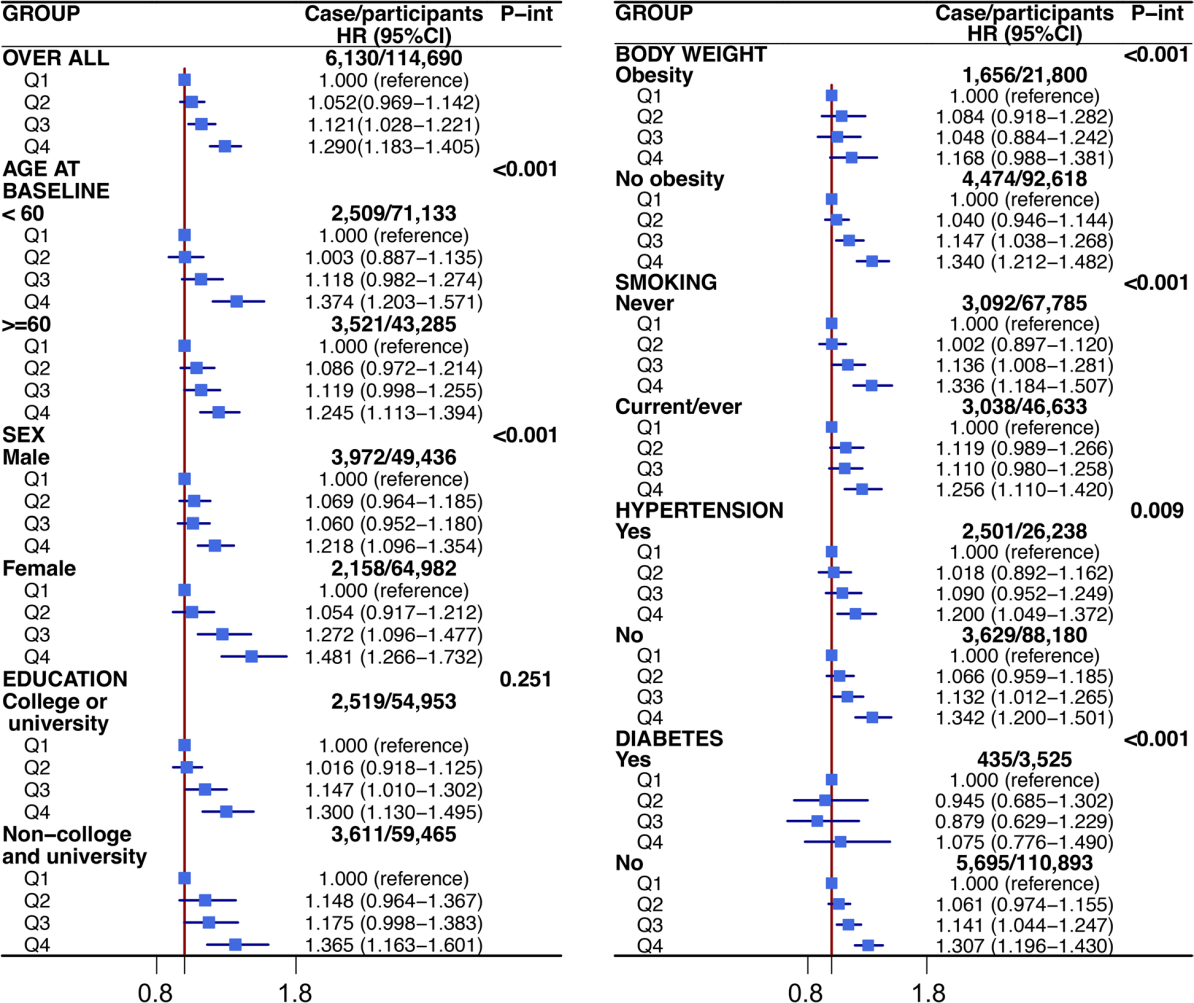
Association of age at job initiation and coronary heart disease stratified by covariates.  Note: Age at job initiation is presented by quantile including Q1 (≥ 22 years), Q2 (21–19 years), Q3 (17–18 years) and Q4 (< 17 years). All analysis were adjusted for all covariates presented in Table  1. *P-int* P for interaction

Several covariates—including age, sex, ethnicity, body weight, smoking status, physical activity at goal, work category, work hours per week, history of meeting a psychiatrist for nerves/anxiety/tension/depression, feelings of worry/anxiety, feelings of loneliness/social isolation, hyperlipemia, hypertension, and diabetes mellitus—showed statistically significant interactions. Subgroup analyses revealed that the associations between age at job initiation and the risk of CHD still remained in most subgroups, with the highest risk of CHD in youngest group (< 17 years old), except for non-white ethnicity, obesity, intermediate diet, job-related shift work, and diabetes mellitus. However, precision decreased in the subgroups because of the smaller sample sizes (Fig. 3; Additional file 1: Figure S3).

### Sensitivity analysis

In our sensitivity analysis (Table 3), we excluded participants who developed CHD within 2 years from follow-up and those with extreme age at job initiation. We considered all-cause death as a competing event and even adjusted for additional risk factors such as family history of CVD, chronic kidney disease, and stress. Despite these variations in the analysis, the association between early job initiation and an elevated risk of CHD consistently remained statistically significant. These robust sensitivity analyses underscore the reliability and resilience of our primary findings.

**Table 3:** The sensitive analysis for association between Age to job initiation with major adverse cardiovascular events

	Age to job initiation (years old)				
Endpoints	≥ 22	19–21	17–18	< 17	*P* for trend
Coronary heart disease					
Sensitive analysis 1	1.00 (Ref)	1.05 (0.97–1.15)	1.14 (1.04–1.24)	1.30 (1.19–1.43)	< 0.001
Sensitive analysis 2	1.00 (Ref)	1.05 (0.97–1.14)	1.12 (1.02–1.22)	1.28 (1.18–1.40)	< 0.001
Sensitive analysis 3	1.00 (Ref)	1.06 (0.97–1.15)	1.08 (1.03–1.13)	1.10 (1.07–1.14)	< 0.001
Sensitive analysis 4	1.00 (Ref)	1.05 (0.96–1.14)	1.11 (1.02–1.21)	1.28 (1.17–1.40)	< 0.001

All sensitivity analyses were performed using Cox regression analysis, adjusting for the covariates presented in Table 1

Sensitivity analysis 1: Participants who had experienced coronary heart disease within 2 years after inclusion were excluded from the analysis.  Sensitivity analysis 2: Participants with age at job initiation less than 9.5 years old (17 – 1.5 times the interquartile range) and greater than 29.5 years old (22 + 1.5 times the interquartile range) were excluded. The interquartile range (IQR) was used to determine the age range for inclusion.  Sensitivity analysis 3: Death was considered a competing risk event rather than a censoring event, and the association of endpoints with age at job initiation groups was assessed using a competing risk model

Sensitivity analysis 4: Additional risk factors associated with coronary heart disease, including family history of cardiovascular disease, chronic stress as reflected by the Patient Health Questionnaire (PHQ)–4 questionnaire score, and chronic kidney disease, were included in the analysis

## Discussion

The study’s main finding is the significant and independent association between the age at which individuals begin their employment and the subsequent risk of CHD. Individuals who commence work at an early age—specifically before the age of 19 years—face a notably higher risk of CHD compared with those who enter the workforce as adults (aged 22 years or older). This observed association remains statistically significant even after adjusting for a wide range of covariates, including socioeconomic status, lifestyle factors, and health-related factors, and demonstrates the robustness of the association. The finding underscores the importance of considering the potential health implications of the timing of labor marker entry.

Notably, the study contributes valuable insights into the largely unexplored relationship between early job initiation and the elevated risk of CHD and provides compelling evidence that starting work at a young age may independently elevate the risk of future CHD. This discovery is especially relevant in the context of the growing number of adolescents who are entering the workforce. Notably, over a quarter of UK adolescents were recently employed [25, 26]. Our findings underscore that it is important that individuals consider an older age (possibly beyond the age of 19 years) for entering the workforce. Notably, early work exposure has been significantly associated with a higher school dropout rate among young people [27]. Our study indirectly highlights the significance of preventing early school dropout and extending vocational exploration into the later stages of life. These measures may mitigate the potential risks to cardiovascular health that are associated with early job entry and promote healthier outcomes for individuals in the long run.

Early exposure to work can have a significant impacts on physical health of adolescent workers, given the highly formative and potentially vulnerable adolescent stage. Although some argue that early employment may positively affect mental health, evidence suggests that such outcomes are largely contingent on job quality, particularly the absence of many stressors [14, 28, 29]. For example, high-quality occupations such as managerial, teaching, clerical, and technical jobs are associated with positive developmental outcomes in adolescents [30, 31]. Importantly, however, such high-quality jobs account for only a small proportion of adolescent employment. The majority of adolescent workers are engaged in low-quality jobs retail and service-industry jobs that are often characterized by low wages and continuous work-related stress [30, 31]. These factors can contribute to an increased risk of future CHD.

A significant proportion of young workers experience a heavy burden from their jobs [32]. Study have reported that approximately 38% of youths in the United States work more than 20 h per week during their school years, and over half of these young workers engage in shift work that commences after 9 p.m. [8]. These working conditions have been associated with unhealthy lifestyles including higher rates of alcohol and cigarette consumption and drug abuse [12, 33, 34]. Our study further reveals that young workers, particularly those who initiate work before 19 years of age, are often more likely be subjected to suboptimal job conditions characterized by low-quality positions and work-related stressors than their adult counterparts (≥ 22 years of age). These conditions can lead to unhealthy behaviors including high rates of substance abuse and smoking and an unhealthy diet, factors that are closely associated with cardiometabolic diseases, and an increased risk of CHD [35, 36]. Moreover, the increased risk of CHD among those who begin work early persists even after accounting for these established risk factors, indicating that other socioeconomic and non-socioeconomic factors may contribute to the association.

Low socioeconomic status, which includes low educational attainment and household income, is a well-established socioeconomic determinant of CHD [37–40]. In our study, participants who commenced their employment before the age of 19 years had lower socioeconomic status, significantly lower educational attainment (college or university degree: 17.7% vs. 89.4%) and lower household income (low income: 17.3% vs. 6.1%) compared with those who entered the workforce as adults. This discrepancy may be attributed to early job entry potentially hindering young individuals from pursuing higher education, a theory supported by studies in the United States, Australia, and New Zealand. These studies concluded that intensive work during school years is linked to decreased college attendance, increased dropout rates, and diminished educational attainment in early adulthood [13, 27, 41]. Furthermore, nationally representative data in Brazil support that adolescents who were not enrolled in school but engaged in work faced an elevated risk of overweight and obesity, which potentially affected the future risk of CHD [42]. Consequently, keeping young individuals in school and encouraging later vocational exploration may mitigate the long-term health risks associated with early job entry.

Although the overall incidence of CHD has decreased given advances in the control of classical risk factors such as smoking, the proportion of hospitalizations among young populations in the United States for myocardial infarction, a CHD subtype, remained at 27% in 1995–1999 and increased to 32% in 2010–2014 [43]. Distinct differences in CHD risk profiles have been recognized between young and old patients [44, 45], with recent studies suggesting that work-related factors such as shift work, night shifts, and stress contribute to the future risk of CHD [5, 18, 46]. Younger workers are more likely to experience work-related stressors and engage in unhealthy behaviors such as smoking, alcohol consumption, and substance abuse than their older counterparts [16, 17, 28]. Our study found that those who started jobs at a younger age were more likely to make poor lifestyle choices such as smoking and keeping an unhealthy diet. It is reasonable to hypothesize that individuals who began work at a younger age are exposed early to work-related risk factors for CHD and this may partly contribute to the increased proportion of young CHD patients. However, this association in young individuals must be confirmed with further prospective studies.

Our study has several strengths, including its prospective cohort design, the long duration of follow-up, and a large number of CHD events from hospital admissions and mortality records rather than from self-reported data. However, some limitations must be considered. First, the observational study design prevents the establishment of a causal relationship between age at job initiation and CHD risk. Second, self-reported age at job initiation may be subject to misreporting, although similar findings were observed when the participants with outlier ages at job initiation were excluded from sensitivity analyses. Finally, the missing values in our study must be acknowledged and may be attributable to participant non-response, data entry errors, and other factors. We specifically encountered missing data in key variables, notably “Work hours per week,” “household income,” and “physical activity at goal,” which are associated with CHD [40, 47, 48]. These missing values may have introduced bias into our findings and impacted the generalizability of our results. To address these limitations, we utilized multiple imputation techniques, methods known for addressing the uncertainty of missing data and producing unbiased results. This approach maximized the available information and included all of the participants. Importantly, however, imputation methods rely on assumptions that may not perfectly capture the true values of missing variables. To advance the understanding of this vital public health issue, further research is needed to expand upon our findings and address these limitations.

## Conclusions

The age at which individuals start their jobs was found to be inversely associated with the risk of CHD. Participants who began working at an age younger than 19 years faced a significantly higher risk of developing CHD compared with those who started working in adulthood (at or after 22 years of age).

### Supplementary Information

**Additional file 1: Table S1.** Definition of coronary heart disease and other baseline comorbidities. Note: ICD=International Classification of Diseases. **Table S2.** The proportion of missing value on baseline covariates. Note: SBP= systolic blood pressure, DBP= diastolic blood pressure. **Table S3.** Baseline participant characteristics in population with and without major adverse cardiovascular diseases. Note: Data are presented by mean (SD) or n (%) for continuous and categorical variables, as appropriate. CHD=coronary heart disease, TDI= Townsend deprivation index, BMI=body mass index, SBP= systolic blood pressure, DBP= diastolic blood pressure. **Table S4.** Association between age at job initiation with major adverse cardiovascular events in multivariate COX regression analysis. Note: Model1 adjusted for age and sex; Model2 adjusted for all of covariates on model1 plus ethnicity, obesity, Townsend deprivation index, household income, college or university degree, smoke status, drinking status, physical activity, diet quality, work category, shift work, work hours per week, seen a psychiatrist for nerves, anxiety, tension or depression, worrier/anxious feeling, sensitivity/hurt feeling, loneliness isolation, systolic blood pressure<120 mmHg, diastolic blood pressure<80 mmHg, hyperlipemia, diabetes mellitus. **Figure S1.** Distribution of age at job initiation of all eligible participants. **Figure S2.** Comparison of cumulative incidence of major adverse cardiovascular events. Note: Kaplan-Meier curves with cumulative hazards of CHD on the basis of the quantile of the age to job started. CHD=coronary heart disease. **Figure S3.** Association of age at job initiation and risk of coronary heart disease stratified by the categorical covariates. Note: Age at job initiation is presented by quantile including Q1 (≥22 years), Q2 (21-19 years), Q3 (17-18 years) and Q4 (<17 years)

## Data Availability

The present study has used data from UK Biobank under application number 91,090. Information detailing how to gain access to UK Biobank can be found at https://www.ukbiobank.ac.uk/.

## Abbreviations

CHD: Coronary heart disease
SBP: Systolic blood pressure
DBP: Diastolic blood pressure
PHQ: Patient health questionnaire
TDI: Townsend Deprivation Index
