# Supplementary material for: Age at job initiation and risk of coronary heart disease: findings from the UK biobank cohort study
Source: BMC Public Health. 2023 Oct 30;23:2123. doi: 10.1186/s12889-023-17034-3 (PMC10614325; doi:10.1186/s12889-023-17034-3)
Supplement: Supplementary file 1 — Additional file 1: Table S1. Definition of coronary heart disease and other baseline comorbidities. Note: ICD=International Classification of Diseases. Table S2. The proportion of missing value on baseline covariates. Note: SBP= systolic blood pressure, DBP= diastolic blood pressure. Table S3. Baseline participant characteristics in population with and without major adverse cardiovascular diseases. Note: Data are presented by mean (SD) or n (%) for continuous and categorical variables, as appropriate. CHD=coronary heart disease, TDI= Townsend deprivation index, BMI=body mass index, SBP= systolic blood pressure, DBP= diastolic blood pressure. Table S4. Association between age at job initiation with major adverse cardiovascular events in multivariate COX regression analysis. Note: Model1 adjusted for age and sex; Model2 adjusted for all of covariates on model1 plus ethnicity, obesity, Townsend deprivation index, household income, college or university degree, smoke status, drinking status, physical activity, diet quality, work category, shift work, work hours per week, seen a psychiatrist for nerves, anxiety, tension or depression, worrier/anxious feeling, sensitivity/hurt feeling, loneliness isolation, systolic blood pressure<120 mmHg, diastolic blood pressure<80 mmHg, hyperlipemia, diabetes mellitus. Figure S1. Distribution of age at job initiation of all eligible participants. Figure S2. Comparison of cumulative incidence of major adverse cardiovascular events. Note: Kaplan-Meier curves with cumulative hazards of CHD on the basis of the quantile of the age to job started. CHD=coronary heart disease. Figure S3. Association of age at job initiation and risk of coronary heart disease stratified by the categorical covariates. Note: Age at job initiation is presented by quantile including Q1 (≥22 years), Q2 (21-19 years), Q3 (17-18 years) and Q4 (<17 years) [file 12889_2023_17034_MOESM1_ESM.docx]

**Table S1: Definition of coronary heart disease and other baseline comorbidities**

| Diagnosis |  | ICD-10 codes | Self-reported diseases (Field ID 20002) | Medication use (Field ID 6177) |
| --- | --- | --- | --- | --- |
| Cardiovascular disease | Coronary heart disease | I20, I21, I22, I23, I24, I25 | 1066, 1074, 1075 | - |
|  | Stroke | I60, I61, I63, I64 | 1081, 1086, 1491 | - |
|  | Heart failure | I50 | 1076 | - |
| Hypertension | - | I10, I15 | 1065, 1072 | Blood pressure medication |
| Hyperlipemia | - | E78 | 1473 | Cholesterol lowering medication |
| Diabetes | - | E10-E14 | 1220, 1222, 1223 | Insulin |
| Chronic kidney disease | - | E10.2, E11.2, E12.2, E13.2, E14.2, I12, I13, N03,  N04, N05, N07, N11, N12, N13, N14, N15, N18, N19 | 1192, 1427, 1519 |  |

**Note:** ICD=International Classification of Diseases

**Table S2. The proportion of missing value on baseline covariates**

|  | Missing value | |
| --- | --- | --- |
| Variables | Cases, n | Proportion,% |
| College or university degree | 932 | 0.8 |
| Household income | 10,426 | 9.1 |
| Obesity | 225 | 0.2 |
| Drinker | 77 | 0.1 |
| Smoker | 231 | 0.2 |
| Physical activity at goal | 16,375 | 14.3 |
| Diet quality | 1,474 | 1.3 |
| Work hours per week | 29,971 | 26.2 |
| Seen a psychiatrist for nerves, anxiety, tension or depression | 318 | 0.3 |
| Worrier / anxious feelings | 2,547 | 2.2 |
| Sensitivity / hurt feelings | 2,962 | 2.6 |
| Loneliness, isolation | 1,331 | 1.2 |
| SBP<120 mmHg | 6,002 | 5.2 |
| DBP<80 mmHg | 6,002 | 5.2 |

Note: SBP= systolic blood pressure, DBP= diastolic blood pressure.

**Table S3. Baseline participant characteristics in population with and without major adverse cardiovascular diseases**

|  | Without CHD | With CHD | P value |
| --- | --- | --- | --- |
| n (%) | 1082,88 (94.64%) | 6,130 (5.36%) |  |
| Age (years) | 55.6 (7.66) | 59.8 (6.42) | <0.001 |
| **Age at job initiation** (years) | 19.6 (3.68) | 18.9 (3.62) | <0.001 |
| Male gender, n (%) | 45,464 (42.0%) | 3,972 (64.8%) | <0.001 |
| White, n (%) | 105,364 (97.3%) | 5,976 (97.5%) | 0.398 |
| TDI | -1.82 (2.75) | -1.91 (2.71) | 0.019 |
| Household income, n (%) |  |  | <0.001 |
| Low | 12,600 (11.6%) | 977 (15.9%) |  |
| Middle | 86,946 (80.3%) | 4,802 (78.3%) |  |
| High | 8,742 (8.07%) | 351 (5.73%) |  |
| Obesity | 20,144 (18.6%) | 1,656 (27.0%) | <0.001 |
| College or university degree, n (%) | 52,434 (48.4%) | 2,519 (41.1%) | <0.001 |
| Drinker, n (%) | 105,379 (97.3%) | 5,976 (97.5%) | 0.435 |
| Smoker, n (%) | 43,595 (40.3%) | 3,038 (49.6%) | 0.001 |
| Physical activity at goal, n (%) | 88,669 (81.9%) | 4,914 (80.2%) | 0.066 |
| Diet quality, n (%) |  |  | <0.001 |
| Healthy diet | 19,022 (17.6%) | 894 (14.6%) |  |
| Intermediate diet | 62,157 (57.4%) | 3,539 (57.7%) |  |
| Unhealthy diet | 27,109 (25.0%) | 1,697 (27.7%) |  |
| Job involved shift work, n (%) | 15,400 (14.2%) | 999 (16.3%) | <0.001 |
| Work category, n (%) |  |  | <0.001 |
| Manager or administrative occupations | 31,438 (29.0%) | 1,539 (25.1%) |  |
| Professional, technical or skilled trades occupations | 59,372 (54.8%) | 3,477 (56.7%) |  |
| Other occupations | 17,478 (16.1%) | 1,114 (18.2%) |  |
| Work hours per week, n (%) |  |  | <0.001 |
| 15 to less-than-20 hours | 1,553 (1.43%) | 54 (0.88%) |  |
| 20 to less-than-30 hours | 4,320 (3.99%) | 176 (2.87%) |  |
| 30 to 40 hours | 68,587 (63.3%) | 3,417 (55.7%) |  |
| Over 40 hours | 33,828 (31.2%) | 2,483 (40.5%) |  |
| Seen a psychiatrist for nerves, anxiety, tension or depression, n (%) | 10,157 (9.38%) | 685 (11.2%) | <0.001 |
| Worrier / anxious feelings, n (%) | 57,892 (53.5%) | 3,182 (51.9%) | 0.018 |
| Sensitivity / hurt feelings, n (%) | 56,663 (52.3%) | 3,105 (50.7%) | 0.011 |
| Loneliness and isolation, n (%) | 15,901 (14.7%) | 901 (14.7%) | 0.99 |
| SBP<120 mmHg, n (%) | 18,517 (17.1%) | 458 (7.47%) | <0.001 |
| DBP<80 mmHg, n (%) | 44,730 (41.3%) | 1,936 (31.6%) | <0.001 |
| Hyperlipemia, n (%) | 12,932 (11.9%) | 1,542 (25.2%) | <0.001 |
| Diabetes mellitus, n (%) | 3,090 (2.85%) | 435 (7.10%) | <0.001 |

**Note:** Data are presented by mean (SD) or n (%) for continuous and categorical variables, as appropriate. CHD=coronary heart disease, TDI= Townsend deprivation index, BMI=body mass index, SBP= systolic blood pressure, DBP= diastolic blood pressure.

**Table S4. Association between age at job initiation with major adverse cardiovascular events in multivariate COX regression analysis**

|  | The age at job initiation (years old) | | |
| --- | --- | --- | --- |
| Endpoints | Cases, n (%) | Adjusted HR(95%CI) | P for trend |
| CHD | 6,130 (5.36%) |  |  |
| Model1 |  | 0.96 (0.95-0.97) | <0.001 |
| Model2 |  | 0.98 (0.97-0.99) | <0.001 |

**Note:** Model1 adjusted for age and sex; Model2 adjusted for all of covariates on model1 plus ethnicity, obesity, Townsend deprivation index, household income, college or university degree, smoke status, drinking status, physical activity, diet quality, work category, shift work, work hours per week, seen a psychiatrist for nerves, anxiety, tension or depression, worrier/anxious feeling, sensitivity/hurt feeling, loneliness isolation, systolic blood pressure<120 mmHg, diastolic blood pressure<80 mmHg, hyperlipemia, diabetes mellitus.

**Figure S1. Distribution of age at job initiation of all eligible participants**

**
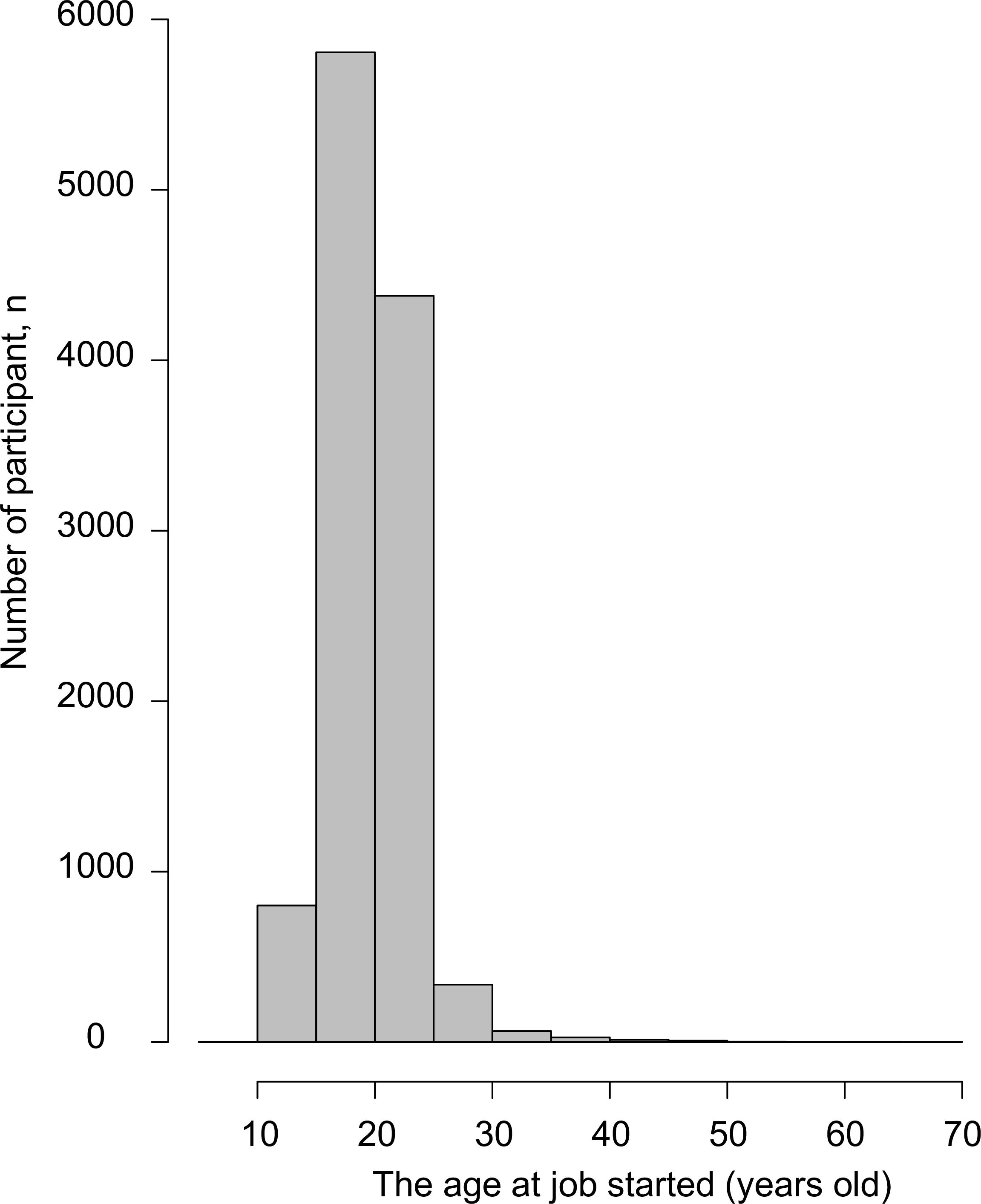
**

**Figure S2. Comparison of cumulative incidence of major adverse cardiovascular events**

**
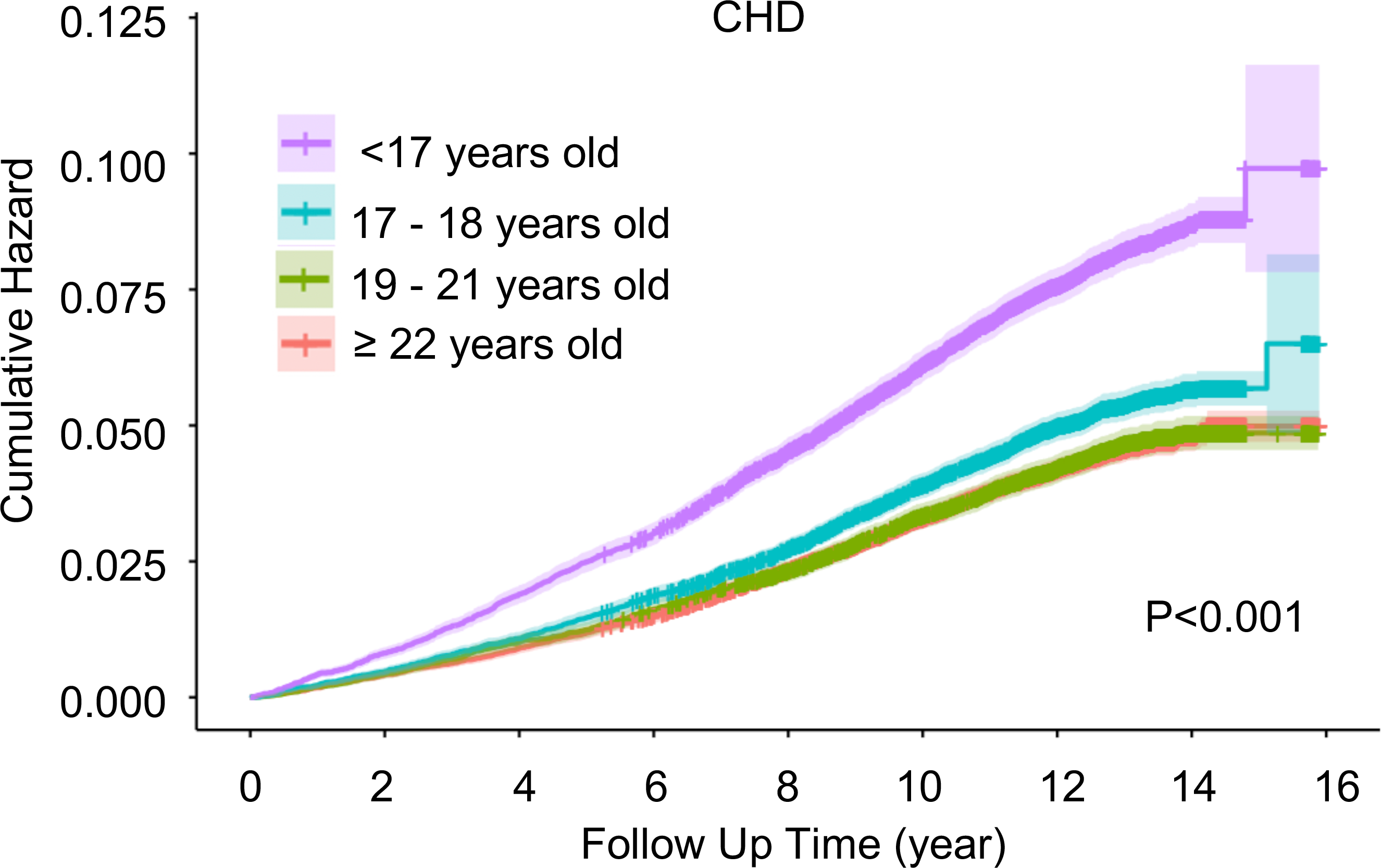
**

**Note:** Kaplan-Meier curves with cumulative hazards of CHD on the basis of the quantile of the age to job started. CHD=coronary heart disease.

**Figure S3. Association of age at job initiation and risk of coronary heart disease stratified by the categorical covariates.**

**Note:** Age at job initiation is presented by quantile including Q1 (≥22 years), Q2 (21-19 years), Q3 (17-18 years) and Q4 (<17 years).
